# Audiovisual time perception is spatially specific

**DOI:** 10.1007/s00221-012-3038-3

**Published:** 2012-02-25

**Authors:** James Heron, Neil W. Roach, James V. M. Hanson, Paul V. McGraw, David Whitaker

**Affiliations:** 1 Bradford School of Optometry and Vision Science, University of Bradford, Bradford, UK; 2 Visual Neuroscience Group, School of Psychology, The University of Nottingham, Nottingham, UK

**Keywords:** Adaptation, Temporal, Recalibration, Spatial, Contextual, Auditory, Visual

## Abstract

Our sensory systems face a daily barrage of auditory and visual signals whose arrival times form a wide range of audiovisual asynchronies. These temporal relationships constitute an important metric for the nervous system when surmising which signals originate from common external events. Internal consistency is known to be aided by sensory adaptation: repeated exposure to consistent asynchrony brings perceived arrival times closer to simultaneity. However, given the diverse nature of our audiovisual environment, functionally useful adaptation would need to be constrained to signals that were generated together. In the current study, we investigate the role of two potential constraining factors: spatial and contextual correspondence. By employing an experimental design that allows independent control of both factors, we show that observers are able to simultaneously adapt to two opposing temporal relationships, provided they are segregated in space. No such recalibration was observed when spatial segregation was replaced by contextual stimulus features (in this case, pitch and spatial frequency). These effects provide support for dedicated asynchrony mechanisms that interact with spatially selective mechanisms early in visual and auditory sensory pathways.

## Introduction

As we navigate through our environment, we routinely encounter a dynamic ensemble of events that produce multiple sensory signals. For example, the task of deciphering human communication requires accurate processing of auditory and visual signals that, whilst generated simultaneously, often arrive asynchronously at their recipient sense organs. This issue is further complicated by the fact that different events often overlap in time and presents a challenge to the nervous system: in order to reap the benefits of audiovisual integration (Bresciani et al. 2006; Koene et al. 2007; Arnold et al. 2010) signals relating to a common cause must be grouped with one another whilst unrelated signals should remain segregated (Gepshtein et al. 2005; Roach et al. 2006; Kording et al. 2007; Colonius and Diederich 2010). A key factor in our perception of these events appears to be the role of recent sensory history. Specifically, with repeated exposure to physically asynchronous multisensory stimulus pairs, observers adaptively recalibrate their perceived onset times. The consequence of this recalibration is to pull the point of subjective simultaneity (PSS—the physical asynchrony that produces perceptual synchrony) towards the adapting temporal interval, rendering physically asynchronous pairs progressively closer to perceptual synchrony (Fujisaki et al. 2004; Vroomen et al. 2004; Heron et al. 2007; Hanson et al. 2008; Harrar and Harris 2008; Heron et al. 2010). Comparable effects have been reported using a variety of multisensory combinations (Keetels and Vroomen 2008; Takahashi et al. 2008; Ley et al. 2009) and within-modality stimulus attributes (Bennett and Westheimer 1985; Okada and Kashino 2003; Arnold and Yarrow 2011).

Conceivably, this mechanism offers functional benefits. For example, audiovisual events placed at different observer-source distances provide asynchronous auditory, and visual signals that would benefit from audiovisual integration yet, on the basis of their perceived onset times, could be deemed to originate from disparate sources. Adaptation to such events has been shown to minimise the perceived asynchrony of these signals, thus promoting the integration of pertinent, co-occurring auditory and visual information (Heron et al. 2007). However, for this effect to have meaningful ecological validity, the recalibration mechanism would need to accommodate heterogeneous recalibration across visual and auditory space. For example, an audiovisual event at 40 m away would require half the recalibration needed for an event 20 m away.

Thus, event-specific adaptation is needed if spurious recalibration is to be avoided. To realise this outcome, the mechanism requires a means of identifying which sensory signals belong to one another. Two likely candidates emerge in the form of spatial and contextual correspondence. A role for the former is given credence by a recent study, showing that the presentation of temporally ambiguous auditory and visual stimuli at disparate spatial locations induces a fixed adapting asynchrony via space-based perceptual grouping (Yarrow et al. 2011). The latter is supported by evidence that high-level contextual correspondence between auditory and visual information (faces and voices) is a powerful driver of temporal recalibration, to the extent that the aftereffects of adaptation follow the adapting contextual arrangement, despite shifts in stimulus location between adapt and test phases (Roseboom and Arnold 2011).

It could be argued that both of these findings are in fact variants of the same hypothesis, with the speech-based grouping representing a higher-level version of space-based grouping. What remains unclear is whether distortions of temporal order are genuinely spatially specific or whether spatial cues simply provide one of many perceptual metrics by which ‘audiovisual objects’ might be constructed and subsequently form the basis of adaptation. Alternatively, a lower-level recalibration mechanism is more likely to be characterised by sensitivity to changes in location relative to visual (De Valois and De Valois 1990), auditory (Cohen and Knudsen 1999) or multisensory receptive fields (King and Palmer 1985; Meredith and Stein 1996), rather than perceptual grouping *per se*.

In the current study, we sought to investigate whether spatial factors hold ‘privileged status’ as drivers of temporal recalibration or, alternatively, whether similar effects could be generated via alternative (higher-level) contextual factors. We adopted a novel approach that allowed the delivery of compelling cues to visual and auditory location, simultaneous adaptation to opposing asynchronies and differing degrees of contextual correspondence between sound and vision. Our results show that adaptation can induce non-uniform temporal recalibrations across external space. However, we proceed to show that this process cannot be replicated with contextually corresponding—but spatially superimposed stimulus pairs. Taken together, these findings suggest that the special specificity of our effects is unlikely to arise from perceptual grouping and points towards the existence of dedicated neural mechanisms for asynchrony perception (Roach et al. 2011) that are sensitive to both spatial and temporal correspondence between auditory and visual signals.

## Methods

### Observers

6 observers participated in the spatial (4 authors, 2 naive) and contextual (3 authors, 3 naive) adaptation conditions.

### Stimuli

The visual stimulus was either a Gaussian blob (σ = 2°, background luminance 50 cd/m², Weber contrast 0.9) presented for two frames (20 ms) at −10° (right) or 10° (left) of a central fixation cross (spatial adaptation conditions) or a horizontally oriented Gabor patch (σ = 2°, background luminance 50 cd/m², carrier spatial frequency of 1 or 4c/deg, Michelson contrast = 0.9) presented at fixation (contextual adaptation conditions). All visual stimuli were presented via a Mitsubishi Diamond Pro 2070 22″ CRT monitor (100 Hz refresh rate, mean luminance 50 cd/m^2^). The exact moment of presentation was controlled by a ViSaGe Visual Stimulus Generator (Cambridge Research Systems, UK), which synchronised presentation to the refresh cycle of the monitor. The auditory stimulus was either a 20 ms burst of bandpass-filtered (200 Hz–12 kHz) white noise (spatial conditions) or a pure tone (auditory frequency of 500 or 2,000 Hz) (contextual conditions). All auditory stimuli were delivered binaurally via Sennheiser HD650 linear headphones. Auditory stimuli were convolved with observer’s individually recorded head-related transfer function (HRTF) representing the spatial offset selected for that trial (+10° or −10°) and presented at 70 dB SPL. This produced compelling auditory locations that were perceptually aligned with the spatial location of the corresponding visual stimulus (for details of the HRTF measurement process see Deas et al. (2008)). The experiment was controlled by custom-written software in MatLab (Mathworks, USA) on a Dell desktop PC. Throughout the experiment, observer’s head position was kept stable via a headrest and fixation was maintained on the centre of the monitor screen. The relative onset times of visual and auditory stimuli were verified via simultaneous capture on a dual-storage oscilloscope.

### Procedures

**Fig. 1:**
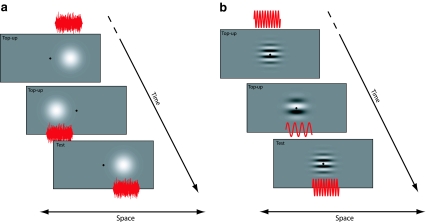
Schematic showing the relationship between the final two (out of six) top-up adaptation presentations and a sample test trial. **a** An example of a spatially incongruent adaptation block where auditory stimuli (*white* noise burst shown in *red*) lead visual stimuli (Gaussian blob) right of fixation, whereas visual stimuli lead auditory stimuli left of fixation. **b** An example of a contextually incongruent condition where high-pitch sounds (pure tone shown in *red*) lead high spatial frequency Gabors and low spatial frequency Gabors lead low-pitch sounds. For both conditions, a sample test trial is shown where vision leads audition

Observers participated in a total of eight adaptation conditions, four spatial and four contextual. All adaptation conditions comprised concurrent adaptation to two audiovisual asynchronies each of which were coupled to a spatial location (spatial conditions) or a pitch/spatial frequency combination (contextual conditions). The four spatial adaptation conditions were categorised as either ‘congruent’ or ‘incongruent’, according to the relative polarities of the asynchronous adapting stimulus pairs. Incongruent conditions involved observers adapting to opposing asynchronies 10° right and left of fixation (as shown in Fig. 1a). These conditions were classified as ‘[AV VA]’ (auditory lead left, visual lead right—as shown in Fig. 1a) or ‘[VA AV]’ (visual lead left, auditory lead right—the reverse situation to that shown in Fig. 1a). Congruent conditions involved observers adapting to either a visual (V) lead 10° right and left of fixation ([VA VA]) or an auditory (A) lead right and left of fixation ([AV AV]). The four contextual conditions saw observers adapting to stimulus combinations where high-pitch tones were coupled with high–spatial frequency (SF) Gabor patches and vice versa (see Stimuli). In all four contextual conditions, visual and auditory stimuli were presented at fixation/straight ahead. Here, incongruent conditions denote situations where the two contextual configurations were assigned opposing asynchronies (e.g. observers simultaneously adapt to a high-pitch tone *leading* a high-SF Gabor and a low-SF Gabor *lagging* a low-pitch tone (as shown in Fig. 1b), or vice versa), and congruent adaptation conditions denote situations where adapting polarity was constant across pitch/SF combinations (e.g. observers simultaneously adapt to a high-SF Gabor *leading* a high-pitch tone and a low-SF Gabor *leading* a low-pitch tone, or vice versa).

### Adaptation phase

For all eight adapting conditions, 120 audiovisual stimulus pairs were presented (60 either side of fixation for the spatial conditions or 60 of each SF/pitch configuration for the contextual conditions) with a fixed 120 ms stimulus onset asynchrony throughout the adaptation period. The laterality of the adapting stimulus pair (spatial conditions) and presentation order of the SF/pitch configurations (contextual conditions) was determined on each trial via random sampling (without replacement). Each adapting stimulus pair was separated by an interval that varied randomly (with a uniform probability) between 500 and 1,000 ms. In keeping with previous work (Heron et al. 2007; Hanson et al. 2008; Heron et al. 2009), observers were instructed to attend to the temporal order of the adapting stimuli but were not required to make perceptual judgments until presented with test stimuli.

### Test phase

**Fig. 2:**
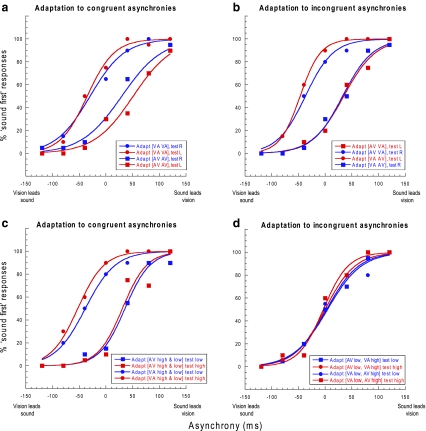
Sample psychometric functions from naive observer CAS showing temporal order judgments (TOJs) as a function of audiovisual asynchrony. These TOJs were made following adaptation to asynchrony where sound led vision (*square symbols*) or vision led sound (*circle symbols*). **a** Spatially congruent conditions where the polarity of the adapting asynchrony was constant across spatial locations. *Red* and *blue curves* represent left and right test locations, respectively. **b** Spatially incongruent conditions where observers adapted to different combinations of opposing asynchronies at different spatial locations. **c** Contextual adaptation conditions. *Red* and *blue* *curves* denote high-pitch/high-SF and low-pitch/low-SF test pairs, respectively. The polarities of the adapting asynchronies were held constant across pitch/SF configurations or **d** were in opposition to each other during adaptation

At the end of the adaptation phase, a two-second pause alerted the observer that the ‘test’ phase was imminent. Each test stimulus pair was prefaced by a top-up phase consisting of six presentations whose asynchronies matched those presented in the adaptation phase. These stimuli alternated either side of fixation (spatial conditions) or between SF/pitch configurations (contextual conditions) with the seventh presentation being the test pair. The laterality/contextual configuration of the test stimulus pairs was determined randomly on a trial-by-trial basis. The test stimuli themselves were presented at one of the seven possible asynchronies: −120, −80, −40, 0 (simultaneous), 40, 80 and 120 ms, which were randomly interleaved within a method of constant stimuli. In the present study, positive asynchronies refer to a physical lead of sound over vision (Fig. 2). Observers made unspeeded, binary forced-choice temporal order judgments (TOJs) as to ‘which modality came first, sound or vision?’ Subjects responded via the computer keyboard, which triggered the next top-up-test cycle. This process was repeated in blocks of 10 repetitions per condition (with a break of at least 30 min between blocks) until a minimum of 30 repetitions had been completed for each of 7 test asynchronies, 2 spatial locations/contextual configurations and 8 adaptation conditions, giving a total of 3,360 trials per data set. Observers completed the 4 spatial conditions in a randomised order before completing the 4 contextual conditions, again in a randomised order.

## Results

For all observers, the percentage of ‘sound-first’ responses for each condition was plotted as a function of test stimulus asynchrony and fitted with a logistic function of the form

$$ y = \frac{100}{{1 + e^{{ - \frac{(x - \mu )}{\theta }}} }} $$

where μ is the audiovisual asynchrony value corresponding to the PSS (the 50% response level on the psychometric function), and θ provides an estimate of temporal order threshold (approximately half the offset between the 27 and 73% response levels). In this way, PSS values were obtained for all observers in all of the conditions.

A representative set of the psychometric functions arising from the spatially congruent conditions is shown in Fig. 2a where the effects of asynchrony adaptation are manifest in the lateral separation between functions of the same colour. For example, comparing TOJs made right of fixation, the proportion of ‘sound-first’ responses is—in relative terms—elevated after adapting to a visual lead left and right of fixation (blue curve, circular symbols) and reduced after adapting to an auditory lead left and right of fixation (blue curve, square symbols). A similar pattern can be observed for TOJs made left of fixation (red curves). As a result, the PSS (the function’s midpoint) is shifted in the direction of the adapting stimulus. This reflects the fact that adapting to a physical lead of one modality over another has the effect of necessitating the same temporal relationship for subsequent test stimuli to appear simultaneous. Figure 2b shows data from the incongruent conditions where observers adapted to opposing asynchronies either side of fixation (e.g. Fig. 1a). If asynchrony adaptation is mediated via a singular, space-insensitive mechanism psychometric functions of the same colour should be superimposed on top of one another—a scenario that is not supported by the effects shown in Fig. 2b. Specifically, TOJs at each test location show that perceived audiovisual timing is distorted in a direction consistent with the polarity of the adapting asynchrony presented at that location. This persists despite the concurrent presentation of an opposing adapting asynchrony 20° away.

In order to compare PSS values across conditions, we calculated ‘Aftereffect magnitude’ (Heron et al. 2010) as the arithmetic difference between PSS values for each adapting polarity, pitch/SF configuration and spatial location:

$$ {\text{Aftereffect magnitude}} = ({\text{PSS}}_{\text{adapt\, A\, leads\, V}} ) - {\text{ (PSS}}_{\text{adapt\, V\, leads\, A}} ). $$

This is equivalent to the lateral separation between psychometric functions such as those shown in Fig. 2 and provides a measure of the overall extent of the temporal recalibration observed in each condition. Thus, values close to zero reflect situations where observers’ TOJs are unaffected by the temporal relationship between the adapting stimulus pairs. For all conditions, aftereffect magnitude values were normalised so that positive values signified repulsive or ‘rebound’ type aftereffects of the type shown in Fig. 2a, b and observed elsewhere in the literature (Fujisaki et al. 2004; Vroomen et al. 2004; Hanson et al. 2008; Harrar and Harris 2008; Takahashi et al. 2008), whilst negative values signified attractive or ‘Bayesian’ type aftereffects (e.g. Miyazaki et al. 2006; Langley et al. 2009).

**Fig. 3:**
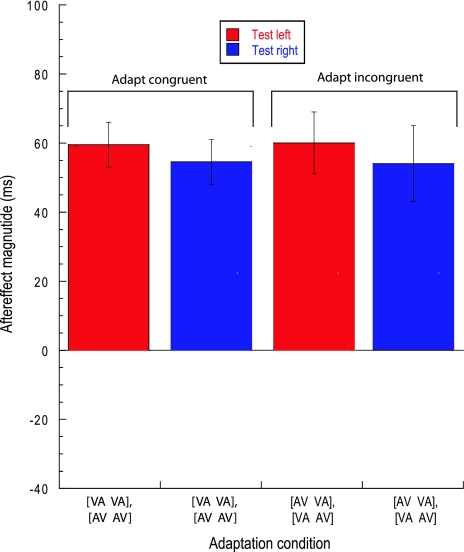
Aftereffect magnitude averaged across observers (*n* = 6) for each of the four spatial adaptation conditions shown in Fig. 2a, b. The height of the *bars* represents the arithmetic difference between PSS values from functions of the same colour (i.e. for a given test location) shown in Fig. 2a, b. Positive values represent repulsive or ‘rebound’ type aftereffects of the type shown in Fig. 2a, b. *Error bars* represent the SEM between observers

Figure 3 plots mean aftereffect magnitude for the spatial adaptation conditions. Congruent conditions represent situations where observers adapted to a common asynchrony polarity at both right and left locations (within an experimental session) whereas incongruent conditions represent situations where observers were exposed to opposing adapting asynchronies at the two test locations (again, within an experimental session). Aftereffect magnitude has been calculated by comparing ‘between-session’ PSS shifts for each test location. Thus, the critical difference between Fig. 3’s congruent and incongruent data is the nature of the concurrently presented adaptation asynchrony presented at the other test location. Fig. 3 confirms that the pattern of results shown in Fig. 2a, b is in agreement with the rest of our observers, namely that adaptation to multiple, opposing temporal relationships promotes a distortion of perceived audiovisual time that is spatially specific. The magnitude of this distortion is equivalent across spatial locations and adapting asynchronies. This pattern of results was confirmed by a repeated-measures ANOVA where aftereffect magnitude showed no dependency on congruency (*F*_1,5_ = 0.014, *P* > 0.1) or test location (*F*_1,5_ = 0.35, *P* > 0.1). The lack of interaction between these factors (*F*_1,5_ = 0.014, *P* > 0.1) highlights a lack of hemispheric specificity: spatial factors promote temporal recalibration with equal efficacy at both spatial locations.

Whilst these effects are consistent with spatially tuned asynchrony perception mechanisms, it is also possible that the effects are simply a product of the perceptual grouping mechanisms discussed earlier (Roseboom and Arnold 2011; Yarrow et al. 2011), with spatial factors providing sufficient cross-modal correspondence between auditory and visual signals. In order to assess the role of perceptual grouping in the effects presented in Fig. 3, we removed spatial cues and replaced them with auditory pitch and visual spatial frequency pairings (see Methods and Fig. 1b for details)—stimulus attributes known to modulate perception by promoting spontaneous mapping across visual and auditory domains (Gallace and Spence 2006; Evans and Treisman 2010). In this experiment, all adapting and test stimuli were presented at fixation. Adapting stimuli could be differentiated on the basis of a consistent temporal relationship between contextually matched pitch/SF pairings (e.g. high-pitch tone always leads a high-SF Gabor patch but a low-pitch tone always lags a low-SF Gabor patch—as per Figure 1B). If perceptual grouping is responsible for Fig. 3’s effects, contextual congruency would be expected to induce comparable effects by allowing observers to form multiple audiovisual ‘objects’ whose perceived timing can be modulated via adaptation. Alternatively, if Fig. 3’s effects arise from genuine spatial specificity, opposing (but spatially co-localised) asynchronies should negate one another during adaptation.

Figure 2c, d shows psychometric functions from a representative observer for the four contextual conditions. As per the spatial conditions, the size of the lateral separation between functions of the same colour reflects the extent of any adaptation-induced temporal recalibration. When adapting asynchrony polarity is held constant across pitch/SF configurations (congruent conditions—Fig. 2c) and TOJs are compared across blocks, the magnitude and direction of the aftereffects are similar to that observed in the spatial adaptation conditions (cf. Fig. 2a, b), irrespective of which asynchrony polarity is coupled with which contextual configuration (i.e. the separation between the blue curves is similar to the separation between the red curves). However, when incongruent adapting asynchronies are interleaved within a block (e.g. Fig. 1b), the effects of adaptation are minimal, as evidenced by the similarity in the lateral position of all curves in Fig. 2d.

**Fig. 4:**
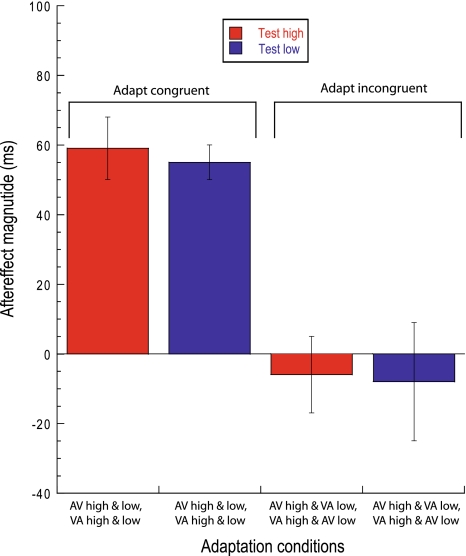
Aftereffect magnitude averaged across observers (*n* = 6) for each of the four contextual adaptation conditions shown in Fig. 2c, d. Aftereffect magnitude expresses PSS shifts for a given test pairing [e.g. test high-pitch/high-SF (*red bars*)]. Congruent conditions denote situations where adapting asynchrony polarity is held constant across pitch/SF pairings. Incongruent conditions denote situations where adapting asynchrony comprises two opposing asynchrony polarities, each coupled with a specific pitch/SF configuration. *Error bars* represent the SEM between observers

Figure 4 plots aftereffect magnitude for all four contextual conditions, averaged across observers. These data confirm the effects shown in Fig. 2c, d: repulsive aftereffects are elicited via adaptation to asynchronies whose polarities are matched across pitch/SF configurations (Fig. 4—‘adapt congruent’ conditions). Conversely, observers are unable to simultaneously adapt to incongruent asynchronies presented at the same spatial location, despite these asynchronies being coupled to compelling (Gallace and Spence 2006; Evans and Treisman 2010), contextually consistent auditory and visual stimulus characteristics (Fig. 4—‘adapt incongruent’ conditions). This effect is reinforced by the highly significant effect of congruency (*F*_1,5_ = 44.4, *P* < 0.01). For both congruent and incongruent conditions, aftereffect magnitude was invariant across high-pitch/high-SF and low-pitch/low-SF test pairs (*F*_1,5_ = 2.46, *P* > 0.1). The slightly negative aftereffect magnitude values observed for the incongruent conditions suggest that rather than inducing the repulsive effects observed thus far, adaptation may generate attractive-type aftereffects (e.g. Miyazaki et al. (2006); Langley et al. (2009)). However, analysis of confidence intervals confirms that both incongruent conditions produced aftereffect magnitude values that were not significantly different from zero.

The data shown in Figs. 3 and 4 were subject to a combined analysis using a repeated-measures analysis of variance with the cue (spatial or contextual) as a between-subjects factor. This revealed a significant effect of congruency (*F*_1,10_ = 22.6, *P* < 0.001) with a highly significant cue × congruency interaction (*F*_1,10_ = 24.2, *P* < 0.001) indicating that the effect of congruency was critically dependent upon whether the cue was a spatial (Fig. 3) or a contextual (Fig. 4) one. Test pairing (left/right or high/low) was not significant (*F*_1,10_ = 0.6, *P* > 0.1). One-sample *t* tests (*df* = 5) confirmed the existence of significant aftereffects in all conditions (*P* < 0.005) except the two incongruent adaptation conditions shown in Fig. 4 (*P* > 0.1).

## Discussion

In the current study, we sought to investigate the role of spatial information in the perceived timing of auditory and visual events. Our results demonstrate two key findings. Firstly, adaptation to incongruent asynchronies promotes temporal recalibration in opposite directions at disparate spatial locations: observers are able to simultaneously hold diametrically opposing perceptions of relative audiovisual time, depending on adapting polarity and spatial location (Figs. 2b and 3—‘adapt incongruent’ conditions). Secondly, when spatial information is replaced with contextual information, observers are unable to exploit linkage between incongruent adapting asynchronies and consistent pitch/SF configurations. Under these conditions (e.g. Fig. 1b), adapting asynchronies fail to instigate temporal recalibration (Figs. 2d and 4—‘adapt incongruent’ conditions).

The results of the current study appear to contradict those of earlier studies where test location was altered between adaptation and test phases (Keetels and Vroomen 2007; Roseboom and Arnold 2011). Keetels and Vroomen found adaptation effects that transferred between two spatial locations. However, a key difference between their study and our own lies in the design of the adaptation phase. In the current study, we employed a paradigm allowing opposing asynchronies to compete for access to the underlying asynchrony mechanism. In contrast, Keetels and Vroomen presented a single adapting asynchrony polarity at a single spatial location during the adaptation phase. Our findings suggest that spatially specific adaptation is only initiated when the presence of multiple audiovisual events make it advantageous to do so. For example, if an observer tracks a single event that translates horizontally across external space, the physical arrival times of its auditory and visual signals will vary little. Under these conditions, veridical perception would be maintained via a common degree of temporal recalibration across space. Conversely, the presence of multiple audiovisual events (e.g. two static events at two different distances from an observer (Heron et al. 2007)) provides an incentive for each of the event’s temporal properties to be independently monitored and—if unchanging over time—recalibrated accordingly.

Roseboom and Arnold (2011) employed a similar approach to that used in the current study and found that temporal recalibration is indeed tied to the characteristics of the adapting stimuli, but argue that contextual linkage between auditory and visual streams drives the specificity of adaptation, as opposed to their spatial location. Specifically, they show that perceptual recalibration of asynchronous auditory and visual speech components follows the identity—rather than position—of the speaker’s face/voice. We found no evidence for recalibration when incongruent asynchronies were coupled with contextually matching stimulus characteristics. On first inspection, this finding appears inconsistent with that of the Roseboom study, perhaps reflecting stronger high-level linkage between faces/voices than our pitch/SF configurations. In the current study, we deliberately chose contextual pairings that—in terms of ecological validity—were relatively arbitrary. Had we employed stimulus characteristics more commonly encountered outside a laboratory setting, Fig. 4’s effects may have shown greater equivalence between congruent and incongruent conditions. However, it is important to note that Roseboom and Arnold’s adapting asynchronies were discriminable via both contextual and spatial cues. As such, an interesting question would be whether their identity-based specificity would persist when both speakers were presented at the same spatial location. Alternatively, differences between the studies may simply reflect the fact that in the current study, both visual and auditory adapting stimuli contained compelling cues to spatial location, whereas Roseboom & Arnold’s auditory stimuli were perceptually directionless.

In summary, we provide evidence for a spatial asynchrony mechanism that facilitates heterogeneous temporal adaptation across external space. Our findings argue against the idea that perceived audiovisual timing is the product of a high-level system that applies global, location-invariant recalibration in response to repeated exposure. Rather, it seems more likely that lower-level, dedicated mechanisms (Ayhan et al. 2009; Bruno et al. 2010; Roach et al. 2011) incorporate both spatial and temporal information when mediating adaptation to the world around us. We have recently shown that asynchrony perception can be modelled as being the product of distributed neural activity across a relatively small number of neurons tuned to different delays (Roach et al. 2011). The results of the current study suggest the operation of independent banks of such neurons, each corresponding to a region of external space and maintaining sensory temporal relationships at that particular location. As mentioned earlier, there appears to be ample neurophysiological evidence of neuronal activity tuned to time, space and sensory pairing (King and Palmer 1985; Meredith and Stein 1986; Meredith et al. 1987; Meredith and Stein 1996). It remains to be seen how the response properties of these neurons can be modified by recent sensory history.
